# Fecal microbiota changes associated with dehorning and castration stress primarily affects light-weight dairy calves

**DOI:** 10.1371/journal.pone.0210203

**Published:** 2019-01-23

**Authors:** Raies A. Mir, Michael D. Kleinhenz, Johann F. Coetzee, Heather K. Allen, Indira T. Kudva

**Affiliations:** 1 Food Safety and Enteric Pathogens Research Unit, National Animal Disease Center, Agricultural Research Service, U S Department of Agriculture, Ames, Iowa; 2 Oak Ridge Institute for Science and Education (ORISE), ARS Research Participation Program, Oak Ridge, Tennessee; 3 Veterinary Diagnostic and Production Animal Medicine, College of Veterinary Medicine, Iowa State University, Ames, Iowa; 4 Department of Anatomy and Physiology, College of Veterinary Medicine, Kansas State University, Manhattan, Kansas; University of Illinois, UNITED STATES

## Abstract

Gastrointestinal tract (GIT) microbiota and stress can impact animal health. Studies have shown that perturbations in the GIT microbiota can influence host health and productivity by affecting physiological homeostasis, metabolism, hematopoiesis and inflammation. The present study aimed to evaluate possible effects of dehorning and castration stress on the GIT microbiota of dairy calves. Dehorning and castration are routinely performed on over 90% of dairy farms, and analgesics like flunixin meglumine (FLU) are given at the time of these procedures to reduce pain. We analyzed fecal microbiota of 24 weaned male dairy calves at two different stages in their life (at 10 weeks for dehorning and 36 weeks age for castration) to determine any GIT microbiota changes due to these stressful procedures and the FLU treatment. Dehorning was performed using an electrocautery dehorner applied to the horn for 10 seconds, and surgical castration was used as the castration method. Our analysis showed that the Shannon diversity index was significantly higher in animals that were not dehorned compared to dehorned animals. Castration stress also resulted in a significant decrease in Shannon diversity index, which was more pronounced in lower weight calves. Body weight and stress had significant effects on the taxonomic profiles of the GIT microbiota. There was a significant difference in the GIT bacterial community structure between heavy- and light-weight calves at Day 3 after castration but not at Day 0 (prior to castration). Our results indicate that dehorning and castration stress reduced microbial diversity of the GIT microbiota, but only in light-weight calves. This work is important for elucidating biological effects of stress on dairy calves and identifying potential modulation points in the microbiota of these food-producing animals to improve animal health and production.

## Introduction

Dairy calves in the US and other developed countries are dehorned to improve the safety of animal handlers, safety of animal-animal interactions, and due to a commercial demand for hornless cattle [1]. Dehorning by hot-iron (cauterization), amputation (scoop, guillotine) and chemical (caustic paste, such as sodium or calcium hydroxide) methods cause immediate and marked pain but is still commonly practiced on 80–94% of dairy and 25% of beef cattle farms in the US [2–4]. A negative impact on growth rate after dehorning, in the absence of any pain relief, was observed in 3 to 6-week-old calves [5]. Although application of suitable analgesic and local anesthetic treatment is recommended, only 12.4% of the US dairy cattle breeders use these agents resulting in pain-induced distress among millions of calves every year [6]. The other management operation commonly employed in the US is castration of male calves to improve meat quality, prevent unwanted mating, and to reduce undesirable aggression and mounting behaviors [7]. Castration is done either by surgical removal of the testicles, burdizzo or clamping techniques to crush the spermatic cords, chemical castration, or rubber bands [8–10]. Irrespective of the technique, castration is a painful and stressful procedure associated with increased plasma cortisol concentration [8], increased mean heart rate [10] and reduced average daily weight gain during 5 weeks following castration [11].

In animals, stress can lead to altered behavior, decreased immune protection and/or altered metabolism (reduced growth and production), or a combination of these responses, all of which affect animal health, productivity and are economically detrimental to the livestock industry [12, 13]. For instance, transportation stress has been shown to increase serum cortisol concentration, decrease rumen pH, alter ruminal bacteria and decrease body weight in cattle [14]. Changes in luminal pH, gut motility, nutrient supplies and host secretions are associated with changes in GIT microbiota [15]. Effects of acute stress on the gastrointestinal tract (GIT) microbiota can be due to the altered eating habits of the stressed calves [9]. Stressed animals also have reduced dry matter intake (DMI), alterations in carbohydrate metabolism, hypoglycemia and increased glucose disposal rates [16–18]. Stress and several factors such as host genotype, diet, ingested microbes, and chemicals can influence the GIT microbiota composition thereby affecting host immunity, nutrient acquisition, disease resistance, metabolism, and the gut-brain axis [14, 19–22]. For example, weaning stress was associated with decrease in abundance of *Bacteroides* genus and increase in *Prevotella* genus in the calf gastrointestinal tract in addition to changing epithelial structure and causing a reduced glucose transport in hindgut [23].

Dysbiosis or an altered GIT microbiota (by infection, antibiotic therapy, stress etc.) can produce metabolites such as amines or indoles which may be toxic to the cells and repress growth [24]. GIT microbiota and intestinal colonization by certain taxa plays a role in determining the body weight or body mass index of the host species [25–27]. For example, obese dogs were found to have a higher abundance of *Proteobacteria* while *Fusobacteria* were more abundant in lean dogs [26]. *Cyanobacteria* YS2 and *Desulfovibrio* were determined to be highly abundant in overweight humans [27]. Also, GIT microbiota in humans and animals has been shown to mature with age until it reaches an adult-like microbiota structure and composition [28, 29]. Animal age has been associated with the taxonomic profiles of the GIT microbiota as seen with a reported higher abundance of *Bacteroidetes* and less of *Firmicutes* in the rumens of pre-weaned calves compared to adult cattle [23, 30, 31].

The NSAID flunixin meglumine, like meloxicam, has been shown to decrease the cortisol response when administered at dehorning and castration [32–34]. Additionally, analgesics have been shown to have a positive impact on leukocyte counts when given at the time of painful and stressful procedures [35]. NSAID have also been shown to affect GIT microbiota compositions in humans and laboratory animals [36–39]; however, its effects on the bovine GIT microbiota are largely unknown. In this study, we analyzed the effects of two acute stressors, dehorning and castration, in the context of NSAID administration and body weight, on the GIT microbiota in dairy calves. Changes in behavior, physiology (plasma cortisol levels, body temperature, heart rate), and neuroendocrine hormones are often used as biomarkers of distress [32, 40–42]. In this study we observed that stress (experienced due to dehorning/castration) in dairy calves was associated with a reduction in GIT microbial diversity and these effects, irrespective of NSAID administration, were more pronounced in light-weight calves.

## Material and methods

### Animal care and management

Standard practices of husbandry and veterinary care, as approved by the Iowa State University Institutional Animal Care and Use Committee (IACUC Protocol #: 6-15-8039-B and 5-15-8016-B) were used in recently published studies [32, 33]. In those studies, calves were group housed with indoor/outdoor access. Pen space per calf met or exceeded the standards set forth in the Guide of the Care and Use of Agricultural Animals in Research and Teaching [43]. The formulated diet met or exceeded recommendations of the National Research Council guidelines for beef cattle. The diet consisted of cracked corn, dried distillers’ grains, a custom protein and mineral supplement, and dry hay. Calves had ad libitum water. Calves were dehorned and castrated using standard industry practices in the United States [32, 33, 40, 44]. Per IACUC protocol, a rescue analgesia protocol was in place for calves experiencing severe pain. Signs of severe pain included excessive lying, reluctance to rise, in appetence, reluctance to move, and/or excessive swelling. No calves met the criteria to require rescue analgesia.

### Exposure to stress, analgesic treatment and sample collection

In the studies referred to above, calves (n = 24) were tagged with a unique identification number and randomly assigned to one of three treatment groups (n = 8 per group) of: 1) topical flunixin meglumine (analgesic) treatment and no stress (SHM group); 2) topical flunixin meglumine treatment and dehorned or castrated (FLU group); and 3) placebo treatment and dehorned or castrated (PLB group) during that study [32, 33].

Dehorning was performed at 10 weeks of age by applying an electrocautery dehorner to the horn for 10 seconds [32]. Sham dehorning was completed using an identical cold dehorner applied to the horn for 10 seconds. Treated calves had transdermal flunixin meglumine (Finadyne Transdermal; MDS Animal Health, UK) applied at the label dose of 3.33 mg/kg concurrently with dehorning as a pour-on along their top-line. Castration was performed at 36 weeks of age using open surgical technique [33]. Briefly, the scrotum of each calf was cleaned with dilute chlorohexidine disinfectant and the lower 1/3 was removed using a new scalpel blade. The testes and spermatic cords were exteriorized by blunt dissection, and the cremaster was broken using manual traction. SHM group calves were subjected to the same handling procedures apart from knife cutting and castration. Pain and stress experienced by the same group of calves was characterized by a combination of four physiological parameters: plasma cortisol, substance P, mechanical nociception threshold (MNT), and skin temperature (Infrared thermography images, IRT) [32, 33].

In the present study, fecal samples collected prior to (Day 0) and at 72 hours (Day 3) after dehorning, castration or sham handling were further analyzed. These fecal samples, collected by rectal palpation into sterile 50 mL conical tubes (Fisher Scientific, USA), and transported on ice to the lab, were processed for DNA extraction using the protocol described in the following section.

### 16S rRNA gene sequencing

We started with samples from 24 calves, but a fecal sample from one calf in the dehorning study (PLB group) was not available, and before the castration study began one calf (SHM group) had been culled for husbandry reasons unrelated to this study; therefore, fecal samples from 23 animals before and after stress were considered for further analysis. This resulted in 46 fecal samples per “stress event” study and a total of 92 fecal samples between the dehorning and castration studies. DNA was extracted from fecal samples using the DNeasy PowerSoil kit (Qiagen, Germantown, MD). DNA yield and purity were evaluated on a Nanodrop (Life Technologies Corp., Grand Island, NY) and by electrophoresis on an 0.8% agarose gel. Previously described primers and conditions were used to amplify and sequence the V4 region of the 16S rRNA gene [45]. PCR reaction mixture contained, 17μl *Accu*Prime *Pfx* SuperMix (Life Technologies Corp., Grand Island, NY), 5.0μM each of the primers, and 25 ng of the template DNA. PCR settings included denaturation at 95°C for 2 min and 22 cycles of (20 seconds at 95°C, 15 seconds at 55°C, 5 min 72°C) amplification followed by final extension at 72°C for 10 min. PCR amplicons were normalized using the SequalPrep Normalization Plate (96) Kit (Applied Biosystems Inc., Foster City, CA). Normalized amplicons were pooled and quantified using Kapa SYBR Fast qPCR (Kapa Biosystems, Wilmington, MA) and sequenced on a MiSeq Instrument using MiSeq Reagent Kit v2 (Illumina, San Diego, CA) following manufacturer’s instructions. DNA from a mock community with defined composition [46] was also used to calculate sequencing error rates.

### Data analysis

For data analysis in the present study, calves were further grouped based on their body weights (light and heavy) in silico. The light-weight group included calves weighing less than 150 lbs at the time of dehorning (average body weight of the light- and heavy-weight groups was 136.6 lbs and 163.4 lbs, respectively) and calves weighing less than 630 lbs at the time of castration (average body weight of the light- and heavy-weight calves was 583.2 lbs and 656.2 lbs, respectively).

The sequences were analyzed in the Microbial Genomics Module (MGM) 1.6.1 (https://www.qiagenbioinformatics.com/solutions/microbial-genomics-solution/) (CLC Genomics Workbench, Qiagen Inc. Redwood City, CA) following the manufacturer’s protocol for clustering of operational taxonomic units (OTUs). Specifically, the paired read data (forward and reverse sequences) were merged to create the highest quality sequences for clustering. The alignment parameters were set as 1 for Mismatch cost, 40 for Minimum score, 4 for Gap cost, and 5 as the Maximum unaligned end mismatches. Then the sequences were trimmed to a fixed length of 250 bp. Samples containing lower than 100 or less than 50% of the median number of sequences were filtered out before using the sequences in OTU clustering. The OTUs were clustered at 97% similarity against the SILVA 16S rRNA small subunit reference database [47] and the metadata were added to the abundance table to aggregate samples based on metadata attributes.

The curated sequences were aligned in the MGM module using MUSCLE by the neighbor joining method and following the Jukes-Cantor model. This alignment was used to create a maximum likelihood phylogenetic tree. The phylogenetic tree and the OTU table describing the taxonomic differences among treatments and between weight groups were used to calculate the Bray-Curtis dissimilarity and generate the PCoA plot. The difference in beta diversity among the treatment groups was analyzed using the Permutation Multivariate Analysis of Variance (PERMANOVA) in the MGM module (CLC Genomics Workbench, Qiagen). This distance-based method tests the association of microbiome composition with any covariates of interest. The analysis and comparisons of alpha-diversity (here represented by measuring Shannon diversity index) between groups was carried out after OTU tables were rarefied to the sample containing the lowest number of sequences (subsampled to 10360 sequences per sample, S1 Fig). Shannon diversity indices were calculated in the MGM 1.6.1 (CLC Genomics Workbench, Qiagen Inc. Redwood City, CA) and compared between light- and heavy-weight calves, before and after stress, using the Student’s T-test. The difference in Shannon diversity index among three treatment groups of calves was analyzed by one-way ANOVA with a cutoff value of 0.05 (p<0.05) used to determine statistical significance using GraphPad Prism (Version 7.0c). To determine the taxa that significantly differ between treatment and weight groups, we used differential abundance analysis (DAA) with the OTU table (at family-level, taxon = family) as input data. Post-hoc test (the false discovery rate, FDR) was used to determine significantly different (FDR *p*-value <0.05) taxa between groups.

## Results

### Association of stress with microbial diversity

**Fig 1:**
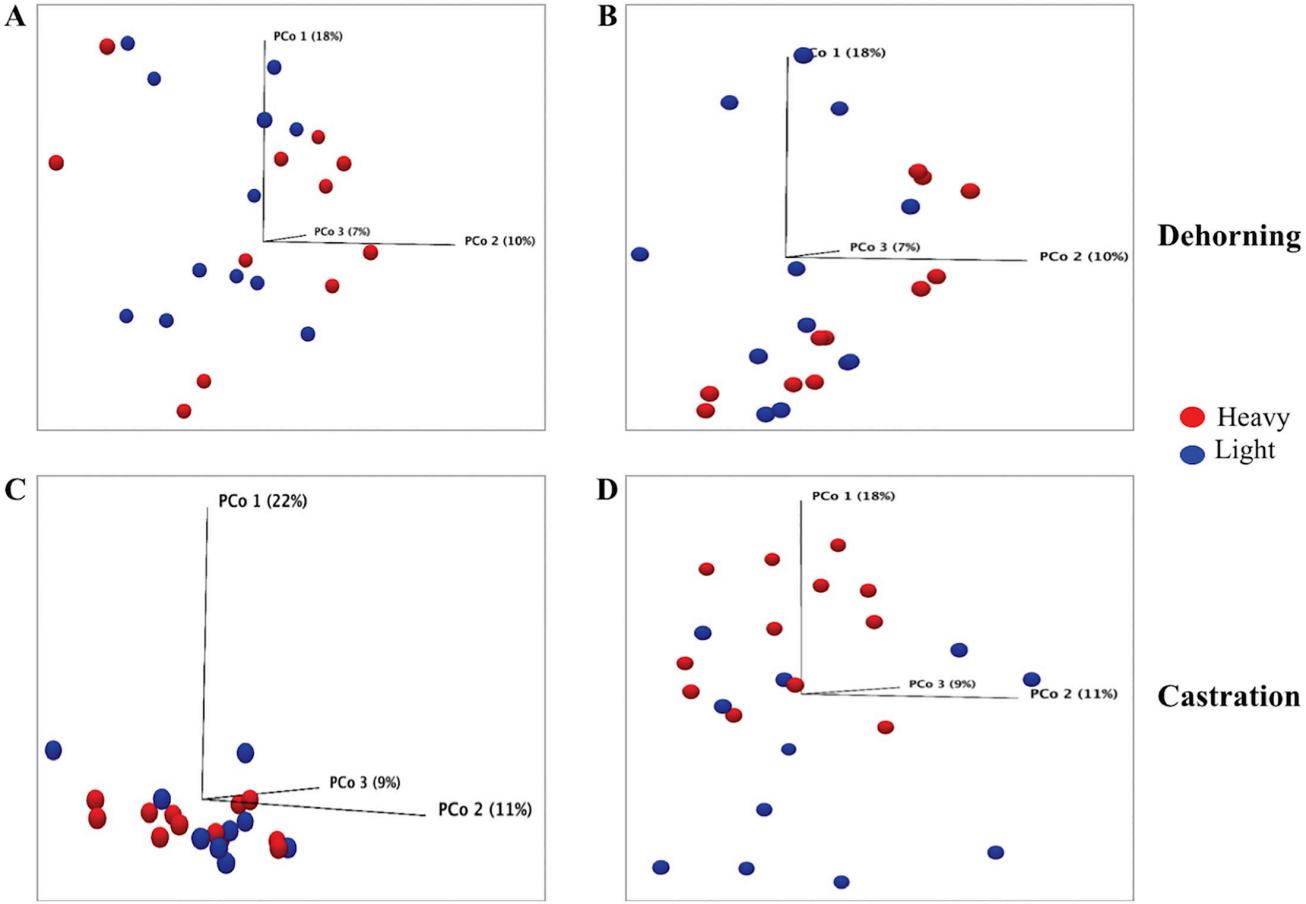
Principal Coordinate Analysis (PCoA) plots for calves, grouped according to body weight, before and after exposure to stress. (A) and (B) represent bacterial diversities in calves before and after dehorning, respectively; (C) and (D) represent bacterial diversities in calves before and after castration, respectively. Each sphere represents one sample. Red spheres represent heavy-weight and blue spheres represent light-weight calves. The input for PCoA was the OTU table containing the rarefied number of sequences observed in each OTU for each sample. The *p-*value obtained in Bray-Curtis distance comparisons was Bonferroni-corrected and a significance threshold of *p*-value < 0.05 was used in all statistical analyses.

Sequencing resulted in 3.58 million reads from 92 samples, which after filtering and removing chimeras yielded 4723 predicted operational taxonomic units (OTUs). This OTU table and the sample information (metadata) was used to determine statistical differences in bacterial community structure among treatment groups over time, as examined via PERMANOVA. No significant differences (*p*-value>0.05) were observed in bacterial community structure among groups due to topical administration of flunixin meglumine (analgesic treatment) (S1 Table, PERMANOVA analysis FLU vs PLB, *p*-value >0.05 at Day 0 and Day 3 of dehorning and castration). Because analgesic treatment did not yield significant differences in community structure, all animals in each experiment were instead grouped and compared by body weight. Bacterial community structure was not significantly different between light- and heavy-weight calves before exposure to either stressors (Day 0, before dehorning or castration), but at Day 3 after castration, there were significant differences in bacterial community structure between heavy- and light-weight calves (Bonferroni-corrected *p*-value <0.05); the difference was not statistically significant at Day 3 after dehorning (Fig 1). The beta-diversity analysis also indicated significant differences in community structure between heavy-weight calves from the SHM group versus heavy-weight calves from FLU or PLB group (*p*-value< 0.05) at Day 3 after stress (dehorning or castration). The difference in bacterial diversity between light-weight calves of SHM versus FLU or PLB groups was statistically significant after castration but not after dehorning (S1 Table). These results suggest that the stressors caused short-term alterations in the GIT bacterial community, and that body weight was a contributing factor to those alterations.

**Fig 2:**
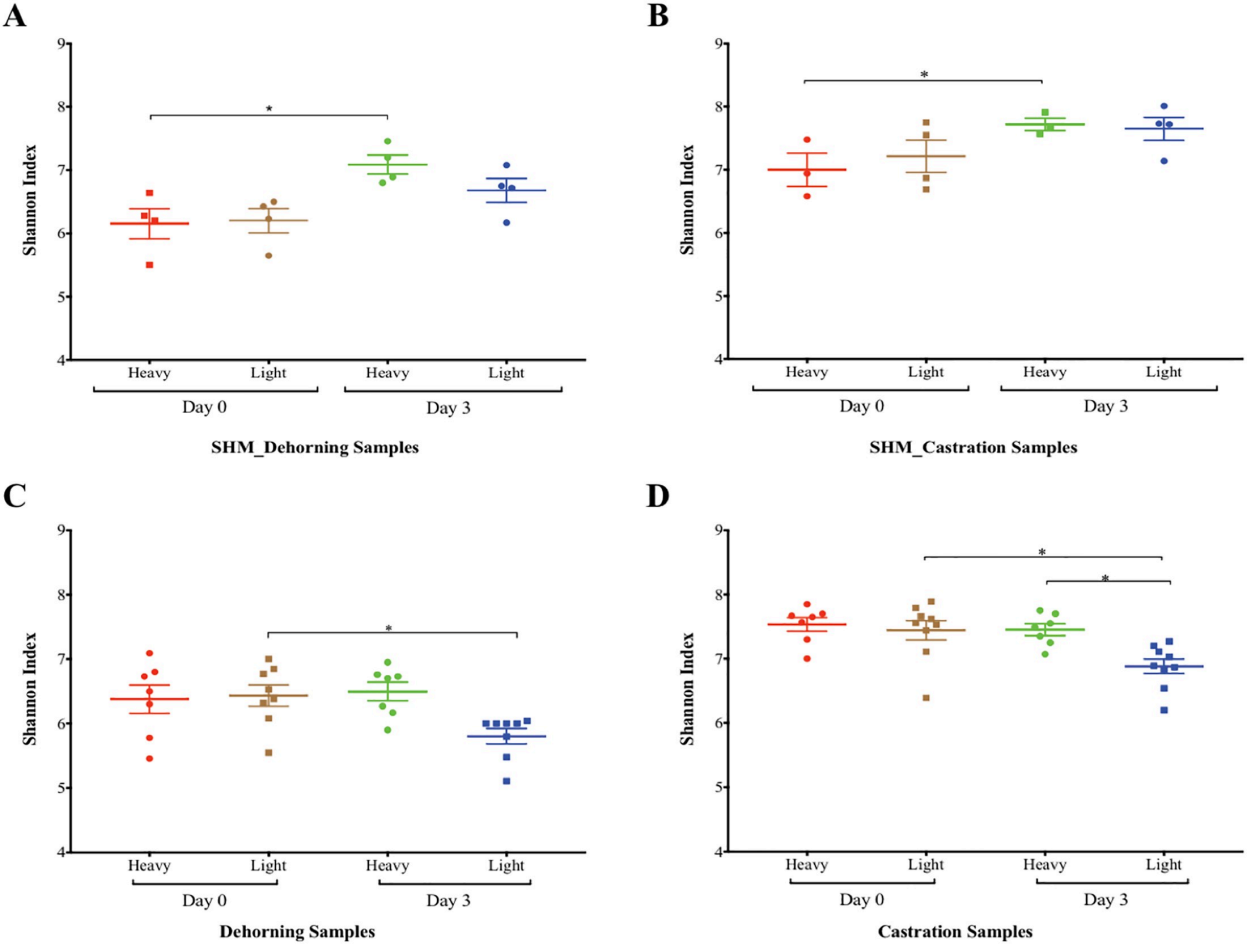
Shannon index before (Day0) and after (Day3) calves were exposed to the sham procedures or stressors (dehorning/castration). (A) and (B), scatter plot of Shannon index (mean ± SEM) for SHM calves (grouped according to body weight) at Day 0 and Day 3 of sham-dehorning (n = 8) and sham-castration (n = 7), respectively. (C) and (D), scatter plot of Shannon index for stressed calves (grouped according to body weight) at Day 0 and Day 3 of dehorning and castration, respectively. Shannon diversity measures were compared between light and heavy calves using the Student’s T-test with a significance threshold of *p*-value < 0.05.

The Shannon diversity index significantly increased from Day 0 to Day 3 of sampling in non-stressed, SHM calves at the time of sham dehorning (Fig 2A) and sham castration (Fig 2B), and the Shannon index was significantly higher in the SHM group compared to stressed groups (*p*-value <0.05, Dunn’s multiple comparison test) at Day 3 after stress (dehorning and castration). The Shannon diversity indices increased in all SHM samples (no stress) regardless of the body weight. In contrast, the GIT bacterial diversity did not increase in dehorned (Fig 2C) and castrated (Fig 2D) calves. In fact, light-weight calves showed a decreased Shannon diversity index following either of the stressors and had significantly lower Shannon index compared to heavy-weight calves after castration (Fig 2D).

### Taxonomic profile of GIT microbiota associated with stress

**Fig 3:**
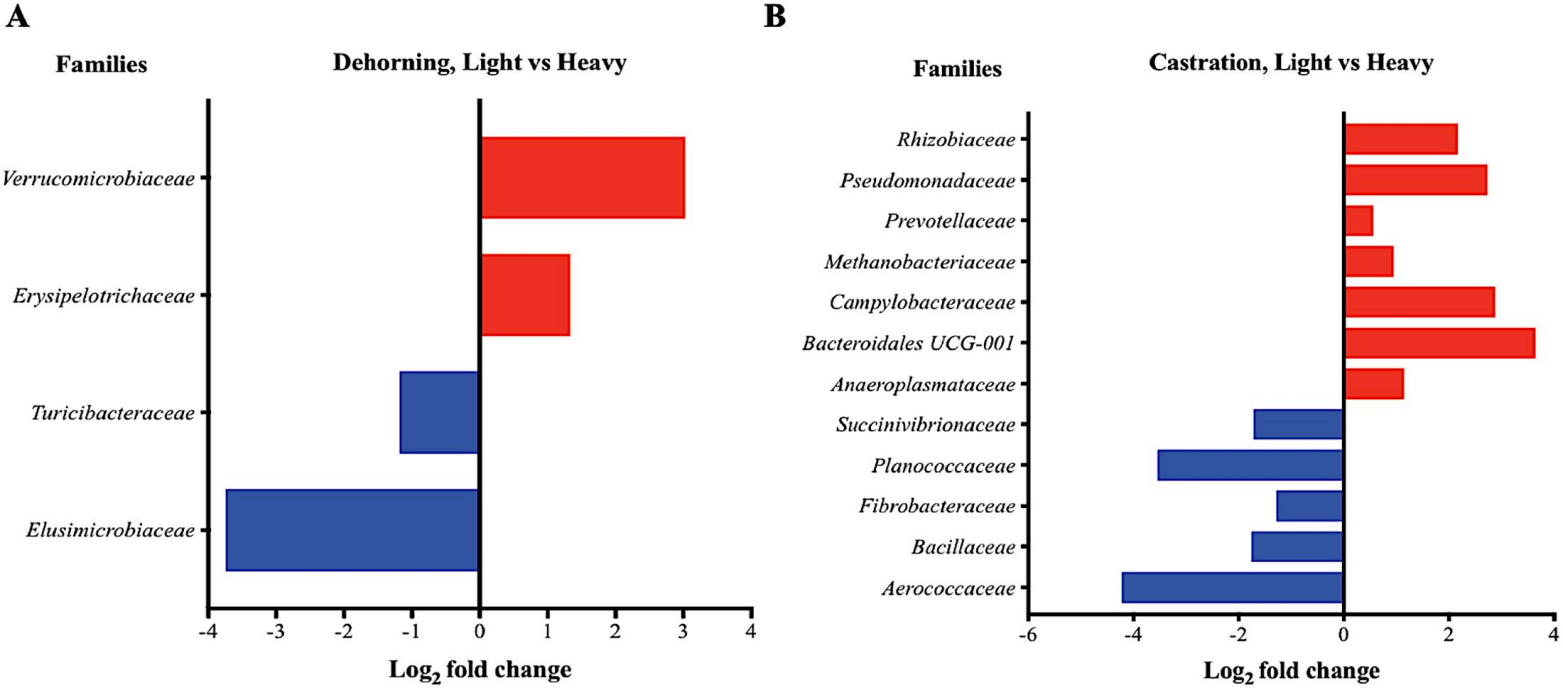
Differential abundance analysis of OTUs (family-level) between heavy- and light-weight calves after dehorning (A) and castration (B). OTU tables were rarefied to the sample containing the lowest number of sequences in each analysis. OTUs were assigned at the family level, and families with relative abundance of more than 0.1% of the total were used in DAA analysis. For multiple comparisons an estimate of the FDR was calculated to determine differentially abundant families with a significance threshold of *p*-value < 0.05. The families represented by the red bars are relatively more abundant in the light-weight calves; the families represented by the blue bars are relatively more abundant in the heavy-weight calves at Day 3 after dehorning (A) and castration (B).

Next, we investigated the taxonomic profile of fecal samples after dehorning and castration to determine the members of bacterial community that were associated with the significant changes in the calf GIT microbiota due to stress. The differential abundance analysis (DAA, on OTU table at family level of taxonomic assignment) indicated association of calf body weight with the taxonomic profile; heavy-weight calves had higher relative abundance of families like *Elucimicrobiaceae* and *Turibacteriaceae*, while light-weight calves had higher abundance of families like *Erysipelotricheae* and *Verrucomicrobiaceae* at Day 3 after dehorning (FDR *p*-value <0.05) (Fig 3A). Heavy-weight calves had higher abundance of *Aerococcaceae* and *Bacillaceae* while light-weight calves had higher *Prevotellaceae* and *Pseudomonadaceae* at Day 3 after castration (FDR *p*-value <0.05) (Fig 3B). Castration was done in older calves (age ~36 weeks), and body weight was a significant determinant of taxonomic profile in animals after castration, which indicates that disturbance in GIT microbiota due to stress may be more pronounced in light-weight calves at this age. Although taxonomic profile before stress was not exactly same between heavy- and light-weight calves, the bacterial community structure was not significantly different. A consistent association of changes in taxonomic profile with the analgesic treatment (flunixin meglumine, topical pour-on application) was not observed in these studies.

### Effect of stress on F: B ratio and abundance of *Proteobacteria*

**Fig 4:**
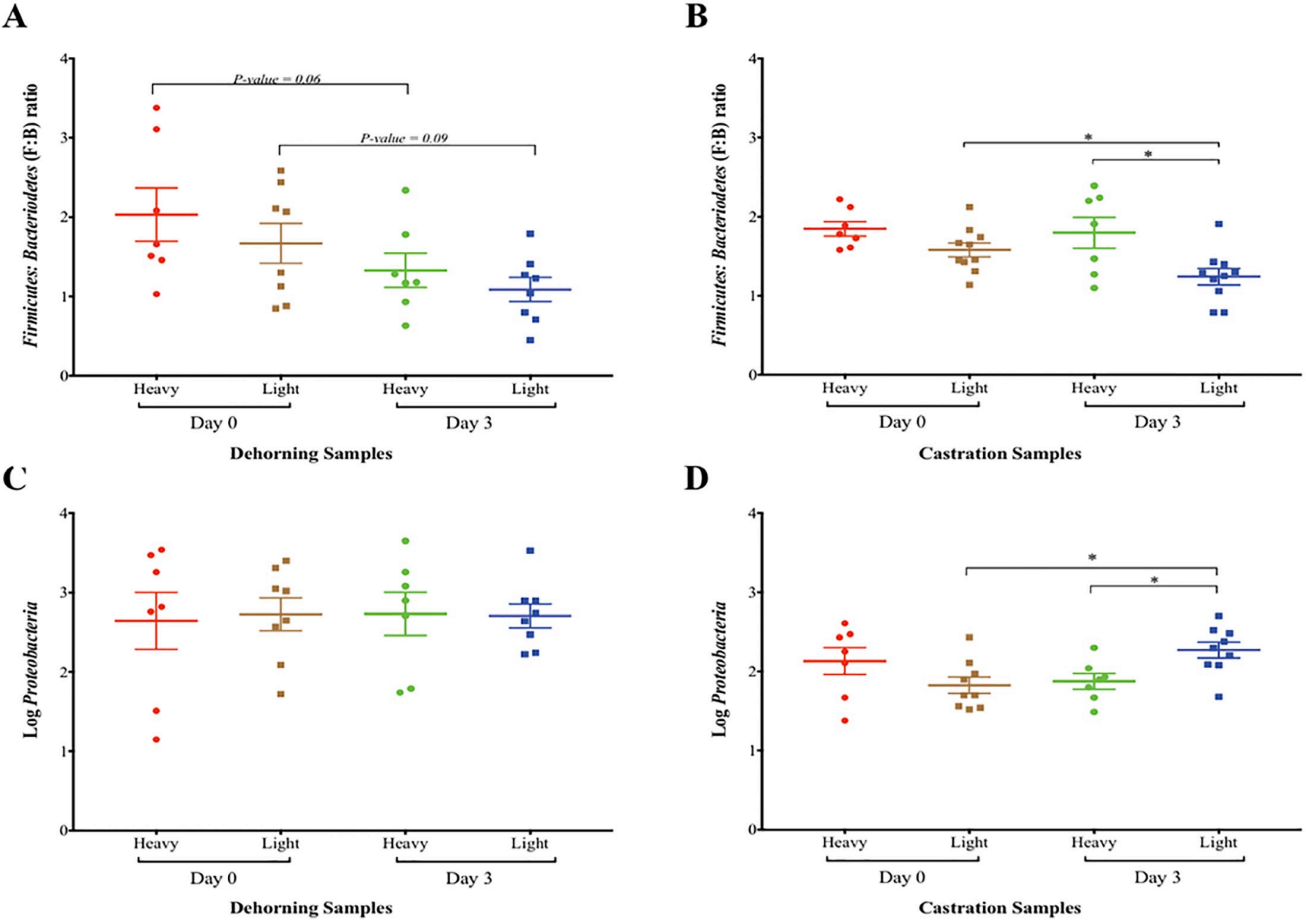
The *Firmicutes*: *Bacteroidetes* (F: B) ratio and concentration of *Proteobacteria* between calf groups (based on body weight) before and after exposure to stress. The F: B ratio was determined by the ratio of total number of sequences representing phyla *Firmicutes* and *Bacteroidetes*, compared among weight groups by ANOVA with a significance threshold of *p*-value < 0.05. The number of sequences representing the phylum *Proteobacteria* in each sample was Log transformed and then compared among weight groups by ANOVA with a significance threshold of *p*-value < 0.05. Statistical analyses and graph preparation were done using the GraphPad Prism (Version 7.0c) software. (A), F: B ratio between heavy- and light-weight calves before (Day 0) and after (Day 3) dehorning. (B), F: B ratio between heavy- and light-weight calves before (Day 0) and after (Day 3) castration. (C), abundance of phylum *Proteobacteria* (Log_10_ number of sequences representing *Proteobacteria*) between heavy- and light-weight calves before (Day 0) and after (Day 3) dehorning. (D), abundance of phylum *Proteobacteria* (Log_10_ number of sequences representing *Proteobacteria*) between heavy- and light-weight calves before (Day 0) and after (Day 3) castration.

The reduction in *Firmicutes* to *Bacteroidetes* (F: B) ratio and increase in *Proteobacteria* has been reported to be an indicator of dysbiosis of the gut microbiota [48, 49]. We observed a decrease in F: B ratio after the calves were exposed to stress, and this decrease was significantly more in light-weight calves (Fig 4A and 4B). There was no significant difference in F: B ratio between heavy- and light-weight calves at Day 0 or Day 3 after dehorning (*p*-value >0.05) but the F: B ratio was significantly lower in light-weight calves after castration (Day 3, *p*-value = 0.027; Fig 4B). Abundance of phylum *Proteobacteria* (measured as Log_10_ of number of OTUs) among all of the samples showed no significant difference from Day 0 to Day 3 after dehorning (*p*-value>0.05) (Fig 4C) and between heavy- and light-weight calves before or after dehorning (*p*-value >0.05) (Fig 4C). In contrast, heavy-weight calves had numerical reduction while light-weight calves had significant increase in the abundance of *Proteobacteria* from Day 0 to Day 3 after castration (Fig 4D).

## Discussion

Previously we reported the effects of dehorning [32] and castration [33] on behavior and pain biomarkers in the same group of dairy calves as used in this study. Altered behavior (or brain function) like weight shifting, impulsiveness, lower mechanical nociception threshold (MNT), higher plasma cortisol was observed in stressed compared to non-stressed, control calves (*p*-value < 0.001) while no significant difference in Substance P or skin temperature (*p*-value > 0.05) due to the stress or NSAID analgesic treatment was observed. In the present study, we evaluated the effect of stress and NSAID on GIT microbiota. Orally administered NSAIDs and antibiotics have been reported to cause marked changes in GIT microbiota in mice and rabbits [36, 37] and metabolites like neurotransmitters and hormones produced by the GIT microbiota have been shown to impact the brain (for example, promote maturation of microglia) [50]. We observed that dehorning and castration stressors primarily affected GIT microbiota of light-weight calves, which showed a significant decrease in Shannon diversity indices in their GIT microbiota compared to sham-treated and heavy-weight calves, irrespective of NSAID administration. In addition, we observed that the changes in microbiota (taxonomic profile) were more pronounced in older calves at the time of castration (36 weeks age).

Dehorning was done at 10 weeks’ age when the animal and GIT microbiota are possibly still maturing [27, 28], which may have masked most of dehorning-related effects. In accordance with other studies examining effect of castration [10, 17, 51], we found the biological effects of stress to be more discernible in older calves at the time of castration, indicating the role of animal age and associated microbiota in detecting responses to stressors. Studies in humans suggest that infants possess a distinct microbial profile and microbial diversity increases with age so that by 2–5 years age, the GIT microbiota is similar to an adult in composition and diversity [29, 52]. Similarly, changes in the GIT microbiota of piglets was associated with weaning; Shannon diversity index of the GIT microbiota increased with age and was not significantly different between 10 and 21 days after weaning suggesting it had reached relatively stable level at these two sampling time points [28].

The changes in microbiota in an animal exposed to a stress may also depend on severity or acuteness of stress and the physiological status of the animal [53]. For example, weaning stress can affect the composition of the microbial community resulting in an undesired population shift and inefficient digestion of feed [31] and a reduction in microbial diversity in the weeks immediately following weaning [23] but these changes can be minimized by proper selection of starter ration, appropriate age of weaning and minimizing other managemental stressors [23, 30, 31]. Cattle that have a high average daily weight gain have been reported to have a calm temperament [54] suggesting that heavier animals could possibly cope with stress better than light-weight animals. Studies in mice have demonstrated increased anxiety-like behaviors and stress induced memory dysfunction due to the disturbance in gut microbiota (by a pathogen or stressor) [55, 56], although such studies have not been validated in dairy calves yet. Stressors like temperature and transportation have been shown to decrease microbial diversity and alter the proportion of bacterial taxa in cattle [14, 57]. We observed similar changes in bacterial taxa following the dehorning and castration stressors. However, further investigation is needed to understand the biological effects of increased proportion of families *Verrucomicrobiaceae* and *Rikenellaceae* at Day 3 after dehorning and castration stress, respectively. Also, *Prevotellaceae* was highly abundant at Day 3, which needs further evaluation because members of this family (for example, *Prevotella albensis*) have been previously shown to relatively increase in stress [14]. Additional studies to evaluate the effects of different routes of analgesic administration, and affirm the biological effects of dehorning and castration stress as observed in this study are being planned.

## Supporting information

S1 Fig Analysis of Miseq sequences. (A) Number of reads obtained per sample from castration (n = 46) and dehorning (n = 46) (B) Rarefaction curves (sub-sampling) for Shannon diversity index (Shannon entropy) for all samples (castration and dehorning samples at Day 0 and Day 3)
(TIFF)

S1 Table PERMANOVA analysis and comparison of GIT bacterial community structure before and after stress. (DOCX)
