# Supplementary figures and images for: Fecal microbiota changes associated with dehorning and castration stress primarily affects light-weight dairy calves
Source: PLoS One. 2019 Jan 23;14(1):e0210203. doi: 10.1371/journal.pone.0210203 (PMC6344101; doi:10.1371/journal.pone.0210203)

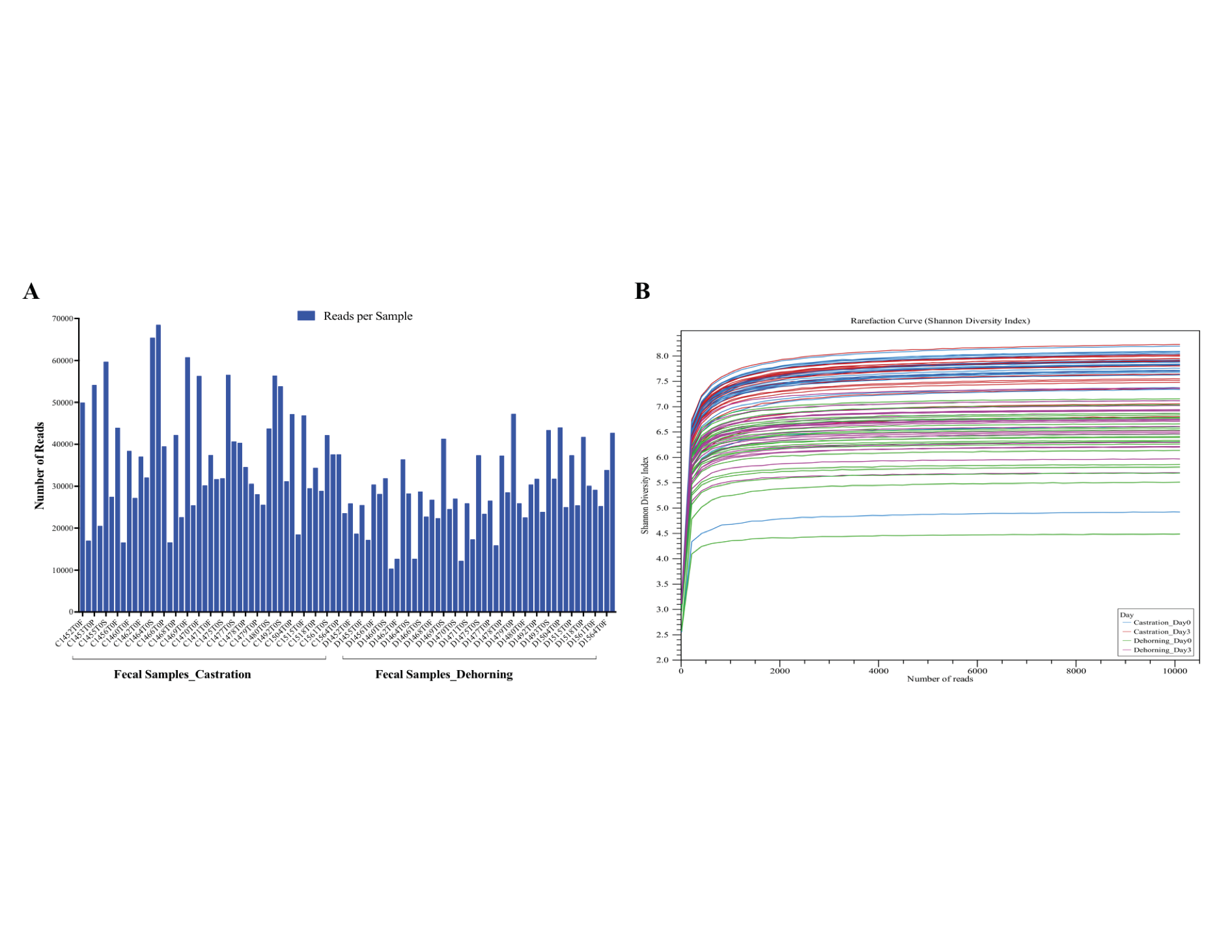

Supplement: S1 Fig — (A) Number of reads obtained per sample from castration (n = 46) and dehorning (n = 46) (B) Rarefaction curves (sub-sampling) for Shannon diversity index (Shannon entropy) for all samples (castration and dehorning samples at Day 0 and Day 3) (TIFF) [file pone.0210203.s001.tiff]
