# Supplementary material for: Fecal microbiota changes associated with dehorning and castration stress primarily affects light-weight dairy calves
Source: PLoS One. 2019 Jan 23;14(1):e0210203. doi: 10.1371/journal.pone.0210203 (PMC6344101; doi:10.1371/journal.pone.0210203)
Supplement: S1 Table — (DOCX) [file pone.0210203.s002.docx]

**S1 Table. PERMANOVA analysis and comparison of the GIT bacterial community structure before and after stress ^a^**

|  |  | Dehorning | | | Castration | | |
| --- | --- | --- | --- | --- | --- | --- | --- |
| Sampling Day | Comparison Groups | Pseudo-F statistic | P-value ^b^ | Pseudo-F statistic | | P-value ^b^ |  |
| Day0 | FLU vs SHM | 1.15 | 0.26 | 0.80 | | 0.91 |  |
| Day0 | PLB vs SHM | 0.61 | 0.91 | 0.90 | | 0.70 |  |
| Day0 | FLU vs PLB | 1.25 | 0.17 | 1.02 | | 0.43 |  |
|  |  |  |  |  | |  |  |
| Day3 | FLU vs SHM | 1.14 | 0.25 | 2.61 | | 0.00 |  |
| Day3 | PLB vs SHM | 0.91 | 0.53 | 3.46 | | 0.00 |  |
| Day3 | FLU vs PLB | 1.22 | 0.28 | 1.02 | | 0.36 |  |
|  |  |  |  |  | |  |  |
| Day0 | SHM-heavy vs FLU-heavy | 1.16 | 0.26 | 1.03 | | 0.43 |  |
| Day0 | SHM-heavy vs PLB-heavy | 0.62 | 0.91 | 1.23 | | 0.27 |  |
| Day0 | SHM-light vs FLU-light | 0.80 | 0.77 | 0.74 | | 1.00 |  |
| Day0 | SHM-light vs PLB-light | 0.89 | 0.51 | 0.83 | | 0.71 |  |
|  |  |  |  |  | |  |  |
| Day3 | SHM-heavy vs FLU-heavy | 2.73 | 0.03 | 1.34 | | 0.17 |  |
| Day3 | SHM-heavy vs PLB-heavy | 1.60 | 0.09 | 1.80 | | 0.04 |  |
| Day3 | SHM-light vs FLU-light | 0.80 | 0.77 | 2.29 | | 0.03 |  |
| Day3 | SHM-light vs PLB-light | 0.66 | 0.86 | 2.26 | | 0.03 |  |

Footnote: ^a^ The GIT bacterial community structure (beta diversity) was compared between treatment groups and between weight groups (PERMANOVA test, F-test) and the p-values obtained from each of the comparisons are shown. Heavy and light refer to two groups of the calves categorized based on the body weight. FLU, PLB and SHM are three treatment groups based on the analgesic administration.

^b^ Comparisons between groups are shown for both the dehorning and castration studies. The sampling days refer to Day0 and Day3 for two studies. Comparisons having p-value <0.05 denote statistical significance at alpha = 0.05.
